# Toward Psychoinformatics: Computer Science Meets Psychology

**DOI:** 10.1155/2016/2983685

**Published:** 2016-06-14

**Authors:** Christian Montag, Éilish Duke, Alexander Markowetz

**Affiliations:** ^1^Institute of Psychology and Education, Ulm University, Ulm, Germany; ^2^Key Laboratory for Neuroinformation/Center for Information in Medicine, School of Life Science and Technology, University of Electronic Science and Technology of China, Chengdu, China; ^3^Department of Psychology, Goldsmiths, University of London, London, UK; ^4^Department of Informatics, University of Bonn, Bonn, Germany

## Abstract

The present paper provides insight into an emerging research discipline called* Psychoinformatics*. In the context of* Psychoinformatics*, we emphasize the cooperation between the disciplines of psychology and computer science in handling large data sets derived from heavily used devices, such as smartphones or online social network sites, in order to shed light on a large number of psychological traits, including personality and mood. New challenges await psychologists in light of the resulting “Big Data” sets, because classic psychological methods will only in part be able to analyze this data derived from ubiquitous mobile devices, as well as other everyday technologies. As a consequence, psychologists must enrich their scientific methods through the inclusion of methods from informatics. The paper provides a brief review of one area of this research field, dealing mainly with social networks and smartphones. Moreover, we highlight how data derived from* Psychoinformatics* can be combined in a meaningful way with data from human neuroscience. We close the paper with some observations of areas for future research and problems that require consideration within this new discipline.

## 1. Introduction

*(1) Current Research Methods in Psychology*. Computer science is poised to have a tremendous impact on psychology. Besides experiments and questionnaires, it establishes a third fundamental research technique: the observation of human-device interaction on a very large scale. It allows psychologists to analyze variables such as personality traits (e.g., extraversion versus introversion), aptitudes (e.g., political), and cognitive functions (e.g., cognitive aging process), as well as behavior (e.g., hazardous driving behavior or active life style). Tracking hundreds of thousands of users, the resulting Big Data requires substantial modeling and cleaning. However, its sheer size in combination with machine learning techniques leverages statistical power (we refer to problems with false positives later on). Most importantly, it avoids most sources of bias, because the behavior of interest is directly recorded. Many biases are inherent to standard psychological measures, for example, the tendency to answer self-report measures in a socially desirable manner (e.g., [1]) or genuine cognitive problems in answering certain questions such as “How many hours do you typically spend on your smartphone?”, an assessment which is strongly undermined by time distortions [2]. Yet, the approach pioneered by Psychoinformatics also poses significant challenges to the two sciences involved. Most importantly, the two must learn to cooperate and ultimately shape an entirely new discipline: Psychoinformatics [3]. Yarkoni [4] describes Psychoinformatics as “…* an emerging discipline that uses tools and techniques from the computer and information sciences to improve the acquisition, organization, and synthesis of psychological data*.” (p. 391).

Traditionally, the psychological sciences rely on two fundamental methods of data collection: experiments and interviews or questionnaires [5]. The former investigate one very particular aspect in a small and entirely controlled setting. The latter assess the broader behavior of a person by means of self-report questionnaire or (potentially structured) interviews [6]. These methods suffer inherent shortcomings. Experiments are usually limited to a single data point (i.e., one experiment) considering a small number of users (who must typically be incentivized to participate). Clearly longitudinal experiments also exist, though these are conducted less frequently due to the high cost and effort involved. Self-report questionnaires and interviews also encounter problems, since humans find it hard to reliably recollect past events, and they are additionally subject to various sources of bias (e.g., the aforementioned tendencies toward social desirability; social desirability refers to the human bias toward presenting oneself in a manner deemed “appropriate” given certain requests or societal norms). In contrast, modern computer science introduces entirely new methods of assessing participants' behavior longitudinally, on large scale, and in comparison to self-reports, in a rather objective manner. Computer science as a discipline is largely concerned with implementing algorithms using computers (or similar devices). For the purpose of this paper, we refer to how algorithms can be used on mobile devices to analyze “Big Data.” Thus, the main aim of the present work on Psychoinformatics is to highlight potential avenues of exploitation of data derived from digital technologies.

*(2) Developments in the Computer Industry Giving Way to Psychoinformatics.* Over the past twenty years, the computer industry has produced a large range of powerful technologies, which have become ubiquitous in everyday life. Smartphones and other mobile devices provide constant connectivity and in doing so have changed our daily lives [7–9]. Together with online platforms such as Facebook, they have become a central venue to communicate, shop, play, or study. As a consequence, digital technologies are pervasive in everyday life and data from such devices could be recorded on a large scale. Finally, cheap hardware allows us to store and analyze large amounts of data at little cost. These new technical innovations provide support for classic psychological methods, such as experiments and questionnaires [10]. First, they enable psychological experiments to be implemented through mobile phones [11]. In the latter study by Dufau et al., the researchers demonstrated the feasibility of conducting experiments on smartphones by implementing a lexical decision task on these devices. As discussed below, this new way of conducting experiments and gathering data needs to be compared with data acquired through classic experimental setups to ensure that data of equal quality can be achieved through Psychoinformatic methods. Is it feasible that neuropsychological tests and other classic test batteries may be implemented on smartphones and be studied not only in patients but also in the broad population? Psychoinformatic experiments can be conducted several times per day over an extended period of time, thus generating a larger number of data points per user. Second, they allow for questionnaires to be administered over mobile phones, potentially asking the participant to contribute data on a daily level, again collecting more data points per user [12]. Here, an interesting variable could be the assessment of mood or the inclusion of experience sampling to assess flow activities in everyday life (the flow concept is explained a bit later in the paper; [13]). The basic shortcomings of both methodologies will, however, remain. Only a limited number of users can be incentivized to regularly conduct an experiment, and questionnaires remain a source of bias (though, of course, self-report inventories will always be of importance in psychology, e.g., to highlight discrepancies between actual recorded behavior and self-view). However, data collection has already benefited from these technologies, for example, easier data processing enabled by the switch from paper-pencil questionnaires to questionnaires administered online, which eliminate errors in recording participants' responses [14]. However, as Psychoinformatics mainly considers variables derived from human-machine interaction on an operation system level (in contrast to filling in “simple” online questionnaires), the data requires significant preparation and preprocessing by skilled computer scientists before they are available for classic inferential statistical analyses. This point is discussed in more detail in the section on data cleaning.

Electric sensors have improved significantly and pose another powerful technology for assessing the condition and behavior of humans. They can measure physical movement (via accelerometers) [15], galvanic skin response [16], or heart-rate (variability) [17, 18]. Over the past ten years, they have become very cost-effective and they require little maintenance by the participant. First, sensors can send their data automatically to a server via a smartphone. Second, efficient processors and powerful batteries have dramatically reduced the need to charge sensors [19]; current fitness trackers, for example, run an entire week on a single charge. The rapid development of technologies gives way to the Internet of Things (IoT), where everyday things such as coffee machines or the fridge are connected to the Internet (see also below) and can serve as data sources.

*(3) The Internet of Things and Psychoinformatics.* As outlined above, the main methodological advantage Psychoinformatics offers over classic psychological techniques is the ability to track human-machine interaction directly on the device. For example, one can track the interaction between a user and their smartphone [20] or (smart) car [21]. This approach can also be extended to online platforms, such as social networks [22] or shopping sites [23]. Data is captured and transferred to a central server for further analysis, without requiring any interaction from the user. Such tracking outperforms traditional methods in terms of both the scale and quality of the data collected. First, it allows researchers to track a very large number of participants, up to hundreds of thousands. Second, it collects numerous data points per day, without demanding anything from the participant. As people increasingly move their lives online, potential data sources become ever richer, ultimately providing more data points per day. Simultaneously, such data sources become ever more plentiful, as our environments become increasingly digital. Soon, we will be able to track interaction with smart cars [24] and coffee machines [25].

This vision of a world, where every device has computational powers and online connectivity, is commonly referred to as “ubiquitous computing.” The term dates back to Mark Weiser's work at Xerox PARC in the 1990s [26]. Meanwhile, it has become mainstream and denotes the corresponding research area in computer science [27]. In an even broader vision, the Internet of Things (IoT) or the Internet of Everything refers to a world, where every item is represented and every process is conducted digitally or at least documented digitally. Necessitating a globally agreed upon set of standards, the IoT thus forms something of a semantic infrastructure. Every device in this world produces data, documenting its actions. The storage and analysis of this data is commonly referred to as Big Data. In this vision, there is no causal relationship between data collection and its analysis; that is, data is commonly analyzed to answer questions that were only vaguely known, if at all, at the time of data collection. Of course, this approach yields the danger of false positive results, particularly in light of the many variables of interest to be gathered via recording of human-machine interaction, resulting in endless opportunities to search for significant correlations. Therefore, independent replication of results observed from Psychoinformatics data sets and carefully designed follow-up experiments (laboratory-based) will be necessary. There are numerous visions of how digitalization may shape our world. As an initial point for further reading, we refer readers to the seminal works by Rifkin [27, 28] and Brynjolfsson and McAfee [29].

*(4) The “Noise” in Big Data.* Admittedly, the “Big Data” collected via Psychoinformatics methods contains a great amount of noise. However, as the methodology generates so much data on so many users, the signal should separate from noise more clearly than ever. For example, take a researcher interested in the investigation of cognitive functions, who wishes to assess cognitive function by studying the changing size of the word pool of a person's language. If the researcher only considers word use across one day, the data set is unlikely to be very representative. Perhaps on this day, the participant only used WhatsApp with his/her child, writing in simple (childish) words. However, by analyzing this person's word use over a longer time window, the standard error of the measure decreases, because digital interactions with a larger number of people can be included in the analysis.

Finally, ubiquitous tracking avoids most sources of bias inherent to questionnaires. Tracking user interaction directly—for example, on a smartphone—remains subject to certain forms of bias (the feeling of being monitored might change the behavior of a person). Yet, these are much less than that present in experiments or questionnaires. Moreover, after a short while, participants should no longer think about the fact that they are being tracked. This clearly needs to be tested empirically, but we can think about this using a highway analogy. If a person moves into an apartment on a noisy street, he/she will clearly be annoyed by the noise for the first few days. After a while, however, the noise is filtered out by the human brain and some people will no longer be aware of it [30, 31]. Of course, there is a big difference between awareness of traffic noise compared with being tracked by another person. Nevertheless, the success story of online social networks such as Facebook demonstrates that a large number of people are not overly concerned about their digital privacy (at least after a while); otherwise, they would reconsider their open profiles, and so forth.

Tracking behavior on the smartphone is likely to lend the greatest insight into human behavior. It captures various aspects of life via a wide range of methods (movement patterns via GPS and text mining to infer mood, communication patterns, and size of the social network) [32, 33]. It is loaded with sensors. It can communicate its data autonomously to a remote server. It serves as the central device to access the web, shop online, communicate with friends, and play games. And, importantly for research budgets, most people already own such a device. According to statista.com [34], in 2016, more than two billion humans will use a smartphone. With this enormous distribution of smartphones worldwide, they are predestined to turn into the most prominent data source for scientists [35].

*(5) The Complexity of Data Cleaning Steps.* The inherently different data characteristics derived from the human-machine interaction require an entirely different mentality from researchers. Big Data, such as that generated by means of ubiquitous tracking, is commonly characterized by the three Vs:* velocity*,* variety*, and* volume* [36]. Data arrives at a very high rate, in various formats and qualities, necessitating substantial means of storage. This data is inherently flawed and dirty. Yet, as indicated above, signal should separate from noise clearly (due to the massive amount of data points collected). While researchers of course need to check up on the collected data (see data cleaning a bit further down below), they must also sacrifice the kind of control they traditionally have in a strict experimental setup. Instead, they need to rely on the statistical power of a large number of measurements.

Frequently, this form of research will rely on data that has been collected for entirely different purposes. For example, a researcher might analyze the logs of a social network. Or they might utilize the billing information of a telecommunication provider. Any such approach, common to Big Data applications, shifts research to post hoc analysis. The scientific question at hand has no influence on the data collection. As a matter of fact, the question might not have arisen at the time the data was collected. This raw data, obtained via diverse applications, requires extensive processing. Initially, it is often cryptic and eludes analysis. It thus necessitates significant modeling before it can be analyzed. Thus, there may be many more processing steps, including various forms of data cleaning. Building models for data analysis will in effect replace a priori experimental design as the “intellectual” challenge in psychological research. This data cleaning processes will largely depend on the unique research question under investigation.

Consider a study on productivity issues in digital work environments. One could hypothesize that because more interruptions are observed, less productivity should be observable, owing to disturbance of the aforementioned experience of flow in one's work. Flow represents a state of high (productive) concentration, in which a person's skill is matched with the difficulty of a task. Smartphones can distract us to a point where reaching a state of flow becomes impossible. The study would thus focus on interruptions due to smartphones in everyday life. Therefore, the computer scientist might model how often a smartphone is flicked on and shut down. This modeling process must thus take many things into consideration. Is it more interesting to assess the length between phone sessions? Or should we calculate the general time spent on a smartphone each day? Should we count time, when the phone is used to listen to music, but not interactively? How should ultrashort smartphone sessions be handled, for example, where the phone's screen is flicked on, but the phone is not unlocked, and there is no further haptic interaction? The precise research question at hand will determine data cleaning and modeling. And any solution will require close interdisciplinary collaboration.

*(6) What Is the Research Agenda of Psychoinformatics?* Naturally, there have been previous collaborative efforts between the areas of psychology and computer science. In particular, Human-Computer Interfaces (HCI) denote the area of computer science concerned with the interaction between users and electronic systems, for example, by means of graphic interfaces or acoustic signals. This research direction thus comprises usability engineering, e-learning, interaction, and information design, among others. Immediately addressing the user, it touches many areas commonly studied by psychologists. In particular, the discipline of affective computing recognizes, reacts to, or mimics human affect [37]. Notably, the HUMAINE project investigated emotion-oriented systems [38]. For an introduction, see http://emotion-research.net/. More narrowly focused, Human-Robot Interaction focuses on the interface between users and (humanoid) robots, thus also touching on aspects of psychology. Both areas are well established within computer science, as documented by the IEEE Transactions on Affective Computing and the ACM/IEEE International Conference on Human-Robot Interaction. Yet, originating in HCI, these areas of research commonly focus on individual users and, up to now, have rarely utilized Big Data technologies. They, too, can thus benefit from the development of Psychoinformatics.

The collaboration between computer science and psychology will finally allow the latter to more practically apply many of their scientific results. Up to now, much quantitative research in psychology could admittedly have enjoyed more practical impact. By this we mean that important research in psychology is conducted in carefully designed laboratory experiments or questionnaire studies, where it is unclear if results can be generalized to real life. Now, however, results from psychology can be validated in everyday life and integrated into the logic of IT systems. Cars will recognize when drivers are sleepy or agitated. Learning software will realize when a student's attention is slipping. Such “affective computing” will be an integral part of most of the devices that surround us [37]. These applications will provide immediate practical value, not only to novel findings but also to many psychological results from previous decades. Of course, this also raises the question of whether Psychoinformatics will create its own unique research agenda. From the literature reviewed in this paper, it becomes clear that Psychoinformatics allows for many psychological research questions to be revisited and tested outside of strict laboratory settings, in everyday life. As mentioned, many important psychological insights have been derived in laboratory settings and therefore testing such results on a wider scale in diverse settings will pose a great challenge. In addition, as with every new interdisciplinary research endeavor, we are convinced that new questions will also arise, extending beyond traditional research questions in both fields. On this point, we present an example from our own work on the Menthal app (see detailed description in the Appendix). When we launched the Menthal project, we aimed to answer the rather simple but important question of how the smartphone dominates our lives. Our custom-made application tracked thousands of smartphones, recording how long participants used their phones each day and which applications are most used (and most distract us from important tasks). Some initial results arising from this project are presented in more detail below. When we analyzed the data set, we became aware of the enormous potential offered by these data; beyond the initial research question, we could indirectly study the sleep behavior of thousands of participants (a previous study from our lab shows that about 36–40% of smartphone users use their smartphone in the last five minutes before going to sleep and in the first five minutes after waking, [39]) or investigate interruptions in everyday life and, therefore, also loss of productivity, even for large populations. Moreover, by considering the GPS signal, it would be possible to combine information from a person's location and smartphone activity with sociodemographic information on the region a person stays in. It is also possible to investigate how the behavior of a person is influenced by the weather at a given moment. In principle, the smartphone data, including its time and location point, could be linked to many external variables. In short, the enormous volume of data from large samples allows the possibility of answering many research questions, which were previously unconsidered. Clearly, this also poses fundamental challenges for ethics committees in determining what can and cannot be studied after the data has been collected. While smartphones and social networks may be an important source for understanding individual's psychological processes, we must also be mindful that these devices are designed for social interaction. Thus, the question arises, to what extent individual processes determined from smartphones truly represent an individual's internal processes or whether this information is influenced by their interactions with others through the smartphone.

*(7) A Short Review of the First Studies in Psychoinformatics.* Currently, the work that falls within the domain of Psychoinformatics is quite scattered. First, it is published in two rather separate scientific communities (psychology and computer science). Second, these are further fragmented in various subcommunities (and different journals), which are not necessarily aware of one another's findings. In general, researchers employ a range of techniques on a variety of data sets, using orthogonal methodologies and pursuing a broad set of research goals.

In recent years, a growing number of studies have been conducted, which broadly fit in the category of Psychoinformatics. The term itself has been independently coined by several workgroups [3, 4]. These studies mainly deal with data sources close to the World Wide Web, such as social networks. We provide a brief review of studies predicting psychological variables from online social networks, such as Facebook, or communication channels, such as Twitter.

In their seminal study, Kosinski et al. [22] investigated over 58,000 Facebook users and demonstrated that it is possible to predict sexuality, ethnicity, or political attitudes from Facebook “Likes” in more than 80% of the cases. This study was also able to predict personality from the Facebook “Likes” (although this was less successful at making predictions on individual level). The prediction accuracy for the Big Five of Personality was between .29 and .43 in this study. Individual differences in personality were assessed with the International Personality Item Pool. A correlation of *r* = .40 suggests that 16% (i.e., *r* = .40^2^) of the variance in Facebook Likes and the personality test overlap. Of interest, correlations of a similar magnitude between smartphone call variables and personality have also been reported [20]. Recently, Kosinski et al. [22] mentioned that this kind of data analysis may be helpful for personalizing web content and online commercials. By studying the communication platform Twitter, Querica et al. [40] observed that influential and popular Twitter users are extraverted and emotionally stable. Extraverted humans can be described as socially outgoing and reward sensitive, optimistic, and sometimes impulsive [41–43]. Qiu et al. [44] reported that personality traits such as neuroticism and agreeableness could also be predicted from tweets. Agreeable humans are likeable people and easily adjust to others [45]. Bai et al. [46] also successfully predicted personality from microblogs (in this case, the Chinese platform Sina). In this study, variables such as number of friends or followers on the microblog were correlated with personality. In sum, a growing number of studies present empirical evidence that data from human-machine-interaction (e.g., Facebook, Twitter, and Sina) can be investigated to successfully predict psychological variables.

Aside from exploiting data from these prominent social media networks, new studies also consider smartphones. In line with the aforementioned studies on Facebook and Twitter, Montag et al. [20] investigated call and SMS variables from smartphones to predict personality traits of smartphone users. While it may appear trivial that extraverts were associated with a range of call variables on the smartphone (as extraverts are socially outgoing, one would expect extraverts to use their phone more), it is noteworthy that Psychoinformatics helps researchers understand which of the large number of call variables on a smartphone is most strongly linked to extraversion. Considering variables such as duration of calls, number of outgoing calls, number of incoming calls, and distinct users called, it becomes clear that this question is not as easy to answer as it initially appears. In the study by Montag et al. [20], the number of outgoing calls was the best predictor for extraversion. An earlier study by Chittaranjan et al. [47] not only linked personality to smartphone variables but also provided a machine learning tool to predict personality from the smartphone variables. Going beyond personality and classic smartphone usage, another recent study provides some initial insights into the relationship between WhatsApp behavior and personality [48]. Again, extraverts reached more out to their social networks (in terms of longer WhatsApp usage). In addition, low conscientious persons stayed longer on WhatsApp [48]. Low conscientious people could be characterized as being less diligent and often not on time. Instead of following their everyday routines, they procrastinate over work tasks and spend too much time on their smartphones. A key advantage of using Psychoinformatics methods to investigate smartphone addiction is highlighted by recent work demonstrating significant time distortion associated with smartphone use, suggesting that smartphone users may be unable to accurately assess the duration of time they spend using their device [2, 49].

Dufau et al. [11] suggest that smartphones can also be used to investigate cognitive variables. Here, it may be possible to observe fluctuations in cognitive functions via the smartphone, which lends itself to the study of cognitive decline in aging societies such as Germany. The study by Dufau et al. [11] is also of relevance from another perspective. In psychology, the terms validity and reliability are central concepts to the quality and generalizability of findings from psychological studies. Before we can consider results from Psychoinformatics alongside evidence collected from classic psychological approaches, whether data gathered from questionnaires via smartphones or similar channels yield the same psychometric properties as data obtained via paper-pencil questionnaires must be systematically tested. Although this is likely (as research has shown that paper-pencil and online questionnaires are comparable with respect to psychometric properties, e.g., [50]), data collected from experiments conducted on smartphones need to be compared with carefully conducted laboratory experiments.

Studies investigating human-machine interaction beyond smartphones or online social networks are rather scarce. Interesting first examples show that the extraction of data from onboard diagnostics (OBD) of cars will be able to identify reckless driving behavior [51, 52] and connecting your fridge to the Internet may help you to follow a healthier diet plan [53].

## 2. Toward Psycho(neuro)informatics

### 2.1. Combining Neuroscientific Data with Data from Psychoinformatics

We have thus far argued for the enhancement of “traditional” psychological data collection by introducing methods from Psychoinformatics. This perspective must also be extended to neuroscience, due to the increasing number of psychologists, who also work in the field of neuroscience [54–56]. Such researchers aim to understand the links between cognition, motivation, and emotion with brain structure and function (and its underlying biochemistry). In recent years, much research has sought to establish links between personality and human brain structure, albeit with heterogenous outcomes [57, 58]. This is also true for molecular genetics [59]. In both fields, problems in replicating results can be linked to differences in preprocessing of imaging data (e.g., MRI), ethnic differences of the participants (both), varying sample sizes, and of course different self-report inventories used to assess individual differences in certain personality traits or related phenotypes (both). Clearly, a central challenge lies in achieving a sufficient sample size (e.g., [60]).

To elucidate these problems, let us consider a number of examples: Trying to understand how individual differences in trait anxiety arise from the human brain, researchers need to choose from the correct neuroscientific tools, as well as from an arsenal of self-report inventories. Unfortunately, many of these self-report measures are only modestly correlated and so the outcome of the research will be highly dependent on the chosen measure of anxiety [61]. Instead of relying exclusively on self-report data, it will be more valid to observe anxiety from human-machine interaction (see below for an example) and link this “real” recorded behavior with variables from neuroscience. The problems of self-report inventories could be significantly reduced by combining observed behavior from Psychoinformatics with neuroscientific data. By applying these methods, real behavior in one study can be made comparable with real behavior in another study. This could lead to better replication of results, as the same dependent variables are investigated. For example, the study by Kern et al. [62] reported that people with high scores on measures of neuroticism tend to use words such as “sick of,” “depression,” “alone,” or “lonely” more frequently on social networks. Thus, quantifying the use of such words in different communications channels by means of text mining would produce an interesting variable to be combined with neuroscientific data. Moreover, personality traits should be reasonably stable across all kinds of different behaviors and diverse situations in everyday life (please see information on the personality paradox by Mischel and Shoda [63]; they describe how stability of personality must be established across different contexts, e.g., a boy behaves in a stable way, shy when being around girls but not shy when he is with a male peer-group), so anxiety may also be reflected in the way we drive cars or our communication patterns via e-mail. Clearly, tracking and use of this data (even for scientific purposes) poses great ethical challenges, which we discuss in the following.

Another example for the importance of the inclusion of real life behavior in neuroscientific research endeavors bases on findings from the study by Bickart et al. [64]. They observed that the size of the amygdala is positively correlated with the size of participants' social network. In this study, the size of the social network was assessed by a self-report questionnaire called Social Network Index (SNI) [64]. In times, where humans carry smartphones with them on a 24/7 basis, not only the sheer number of contacts saved in their smartphone but also the activity of their social network in terms of incoming/outgoing calls will provide a more precise picture of their social network size and activities.

Aside from this research on personality and social networks, a large number of research topics such as mood or wellbeing can also benefit from the inclusion of Psychoinformatics methods. The methodology thus continues the tradition of the experience sampling method (ESM), which has been used in psychology for many years. In such paradigms, participants wear a tracker in everyday life and are asked at random intervals what they are doing and feeling over the course of the day. The ultimate aim of Psychoinformatics, however, is that participants will no longer be asked directly, as these questions can be answered by the data from human-machine interaction. This would be least invasive for the participant. Of course, much neuroscientific research will always depend on strictly controlled experimental conditions. This is particularly true for imaging studies, such as those using MRI. On the other hand, mobile EEG systems are already in existence and have been used to record brain activity in environments such as zero-gravity [65] and other more natural settings [66]. In addition, biological variables such as cortisol measures or genetic samples can be collected in the field with relative ease and can be combined with data derived from Psychoinformatics (e.g., [67]). In short, neuroscientific techniques differ strongly in their applicability to be included in the field outside the laboratory.

**Figure 1:**
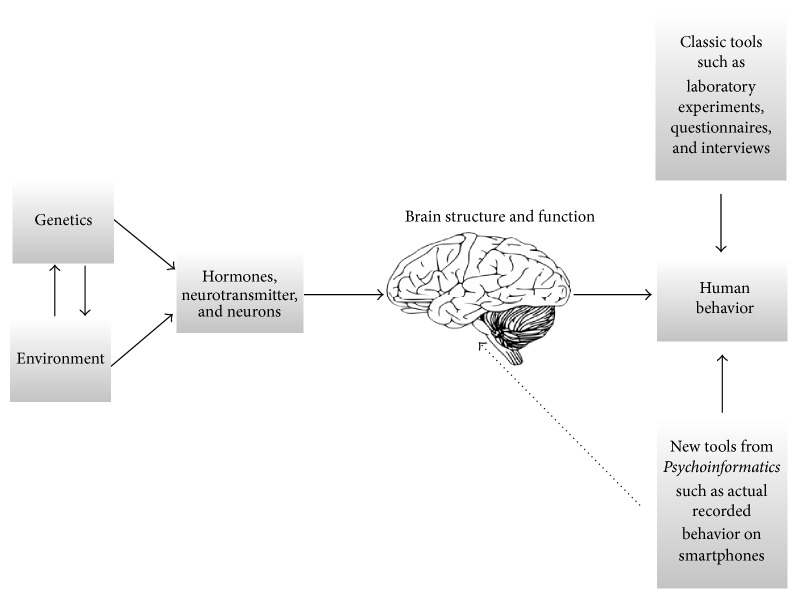
The inclusion of tools from Psychoinformatics will add a new interesting layer to neuroscientific psychological work (the depicted brain has been taken from https://pixabay.com/; Public Domain).

Psychologists may also be wary of sacrificing their long established methods to rapidly evolving technologies. We argue that this will not be the case. Again, self-report, classic lab-based experiments or interviews will not be eliminated but rather compared and enhanced with what can be objectively observed. In particular, biases in one's own perception and actual recorded behavior will make it possible to add a new layer to both research and counseling. In this context, we strongly believe that* Psycho(neuro)informatics* will also have an impact on behavioral neuroscience.  Figure 1 illustrates this relationship in more detail. This figure shows that genes and the environment interact and shape hormone and neurotransmitter levels [68, 69]. In the future, more and more studies will also investigate how the environment can influence methylation patterns and make genetic information available. Again, the measure of environmental variables can be enhanced by incorporating real recorded behavior from everyday life. Following from this, we are of the opinion that the growing field of epigenetics can also profit from the inclusion of methods from Psychoinformatics [70]. All in all, molecular genetics, epigenetics, hormones, neurotransmitter systems, neurons, and so forth represent the biochemical foundations of brain structure and function. Individual differences in the structure and function of the human brain might then be able to explain individual differences in personality traits and other related psychological variables. Finally, we include a dashed line in the figure. This line refers to the possibility of applying machine learning algorithms to neuroscientific data. This is already common practice and so we will not discuss the point further here but refer readers to the work of Nouretdinov et al. [71] and Pereira et al. [72]. Thus, we argue that Psychoinformatics must be incorporated into the assessment of human behavior, as such recorded behavior may be more closely linked to our biology than self-report assessments. Future research can establish whether this assumption is correct.

### 2.2. Challenges

The core challenge for* Psycho(neuro)informatics* lies in its interdisciplinarity. Neither psychology nor computer science can achieve this level of progress independently. Psychologists lack the ability to construct large-scale tracking systems and to manage the resulting data. Thus, they stand to benefit from methods of data modeling and mining. Computer scientists, on the other hand, lack the domain expertise, as well as the long tradition of (ethically sound) research on human subjects. Both sciences have yet to establish common ground, a canonical approach, terminology, and methodology.

Accordingly, both sciences need to cultivate a common research culture. Currently, results in computer science are largely published at large conferences; journal articles frequently only extend previous conference publications. Psychology on the other hand publishes predominantly in (equally peer-reviewed) journals. Hence, both sciences have a different speed of publication. Similarly, universities must adapt to interdisciplinary research undertakings. They need to support careers that are not particularly advanced inside computer science but conduct groundbreaking research in collaboration with psychologists. Or they need to establish corresponding degree programs and departments. Equally, funding agencies need to be open to interdisciplinary applications.

As with any technological paradigm shift, there are ethical challenges to be addressed. Naturally, data privacy is a major concern. However, psychological research has dealt with private and intimate data since its inception and has an established code of conduct for handling data, which can be readily adapted to include digital data. More problematic issues arise, when psychological findings are put to practice in Big Data applications. One might deduce personality features of a user from his online behavior and hence have the potential to deny him/her a particular job. Or one might be able to assess the emotions of an online shopper and “bait” the individual accordingly. While these questions must be addressed, they will become part of a wider discussion regarding the use of Big Data technologies. Additionally, further ethical issues must be expected to arise over the coming years. In particular, different political systems might handle data protection issues in a different way.

Finally, the scientific community has to address data access as a new factor influencing the work of researchers. Today, many publications require scientists to disclose industry funding. After all, such a relationship may result in a conflict of interests and, in the worst case, could influence research or results. Given the novel methodologies, access to proprietary data is an equally important factor of large corporations to hand out “favors”: For example, given access to a large social network, a scientist may be able to discover and publish an entire range of findings, but this may be impeded if the company deems these findings controversial. It must, therefore, become mandatory for scientists to disclose proprietary access to data from any external source that might trigger a conflict of interests.

While Psychoinformatics is still in its infancy and may not even be recognized as such, the path ahead is clearly laid out. Over the next decade, we will see numerous and massive research undertakings between psychology and computer science. The sooner the research community realizes that these efforts are not singular events but part of a paradigm shift, the sooner the two sciences can establish common ground, canonical methodologies, and taxonomies, as well as common ethical standards. And, eventually, this novel research direction will establish a field of its own.

### 2.3. Conclusions

The next decade will see an increasing number of research undertakings, residing squarely between computer science and psychology. Most might not be coined as Psychoinformatics. Many might not involve traditionally trained computer scientists or psychologists. Some might not even be aware that they are pursuing a psychological question. Yet, intentional or not, computer science will, to some degree, change the basic methodologies in psychology.

## Figures and Tables

**Figure 2:**
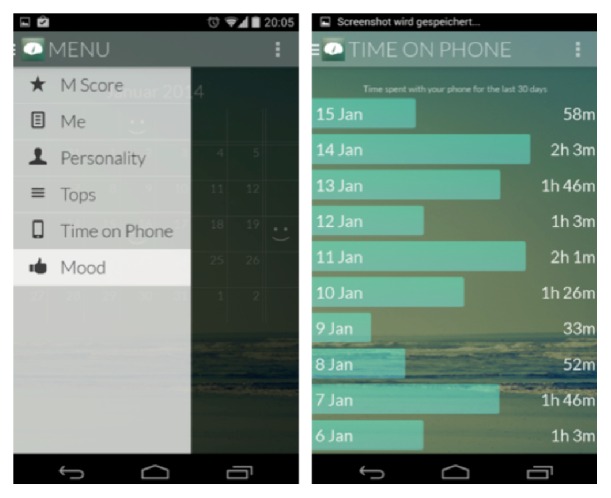
Screenshots of our application “Menthal.”

## Appendix

### Menthal as an Example for a Large-Scale Project in Psychoinformatics

In the following, we would like to give insights into our own “Menthal” project (Mental Health Diagnostics, https://menthal.org/). This project illustrates how a study in Psychoinformatics develops and can be conducted.

Menthal assesses smartphone usage on a very large scale. In less than ten years, smartphones have dramatically altered how we communicate, navigate, date, play, and travel. The resulting changes in our society are evident, yet they have not been scientifically studied. We wanted to log how people actually spend their time on the phone. This behavior was to be assessed directly via the phone, objectively, without relying on self-reports. The question remained: how to incentivize large numbers of users to provide insight into their phone behavior.

To attract users, we developed an app that tracks users' smartphone usage (see Figure 2). It informs users how long they use their phone, how often they flick on their phones, and which apps are most prominent in one's own user history, and so forth. Smartphone users can then decide if their phone behavior is questionably high and track progress on reducing it. On a technical level, the app sends raw data (e.g., “phone unlocked” and “app started”) to our server. The latter computes the corresponding aggregate functions (e.g., how long and how often) and returns this information back to the users' phones in a visual manner. This data also remains on our servers for scientific analysis. In essence, we thus copy the business model of Google: we provide a free and useful service; in return, the users contribute their data. We communicate the approach openly via an informed consent form completed by the participant during app installation.

This application also offers participants the option of contributing additional information on their personality or daily mood. By providing us with this information, they again receive feedback on their personality scores or see how their mood changes over time (mood diary) to incentivize participation.
